# ICTV Virus Taxonomy Profile: *Botourmiaviridae*

**DOI:** 10.1099/jgv.0.001409

**Published:** 2020-04-24

**Authors:** María A. Ayllón, Massimo Turina, Jiatao Xie, Luca Nerva, Shin-Yi Lee Marzano, Livia Donaire, Daohong Jiang, ICTV Report Consortium

**Affiliations:** ^1^​ Centro de Biotecnología y Genómica de Plantas, CBGP (UPM-INIA), and Dpto. Biotecnología-Biología Vegetal, ETSIAAB, Universidad Politécnica de Madrid, Madrid, Spain; ^2^​ Institute for Sustainable Plant Protection – CNR, Torino, Italy; ^3^​ Plant Pathology Department, College of Plant Science and Technology, Huazhong Agricultural University, Wuhan, PR China; ^4^​ CREA – Research Centre for Viticulture and Enology, Congegliano, Italy; ^5^​ Department of Biology and Microbiology, Department of Agronomy, Horticulture, and Plant Sciences, South Dakota State University, Brookings, SD, USA; ^6^​ Biology of Stress and Plant Pathology Department, CEBAS-CSIC, Murcia, Spain; ^7^​ The State Key Laboratoryof Agricultural Microbiology, College of Plant Science and Technology, Huazhong Agricultural University, Wuhan, PR China

**Keywords:** *Botourmiaviridae*, ICTV Report, taxonomy

## Abstract

The family *Botourmiaviridae* includes viruses infecting plants and filamentous fungi containing a positive-sense, ssRNA genome that can be mono- or multi-segmented. Genera in the family include: *Ourmiavirus* (plant viruses), and *Botoulivirus*, *Magoulivirus* and *Scleroulivirus* (fungal viruses). This is a summary of the International Committee on Taxonomy of Viruses (ICTV) Report on the taxonomy of the family *Botourmiaviridae*, which is available at ictv.global/report/botourmiaviridae.

## Virion

**Fig. 1.**
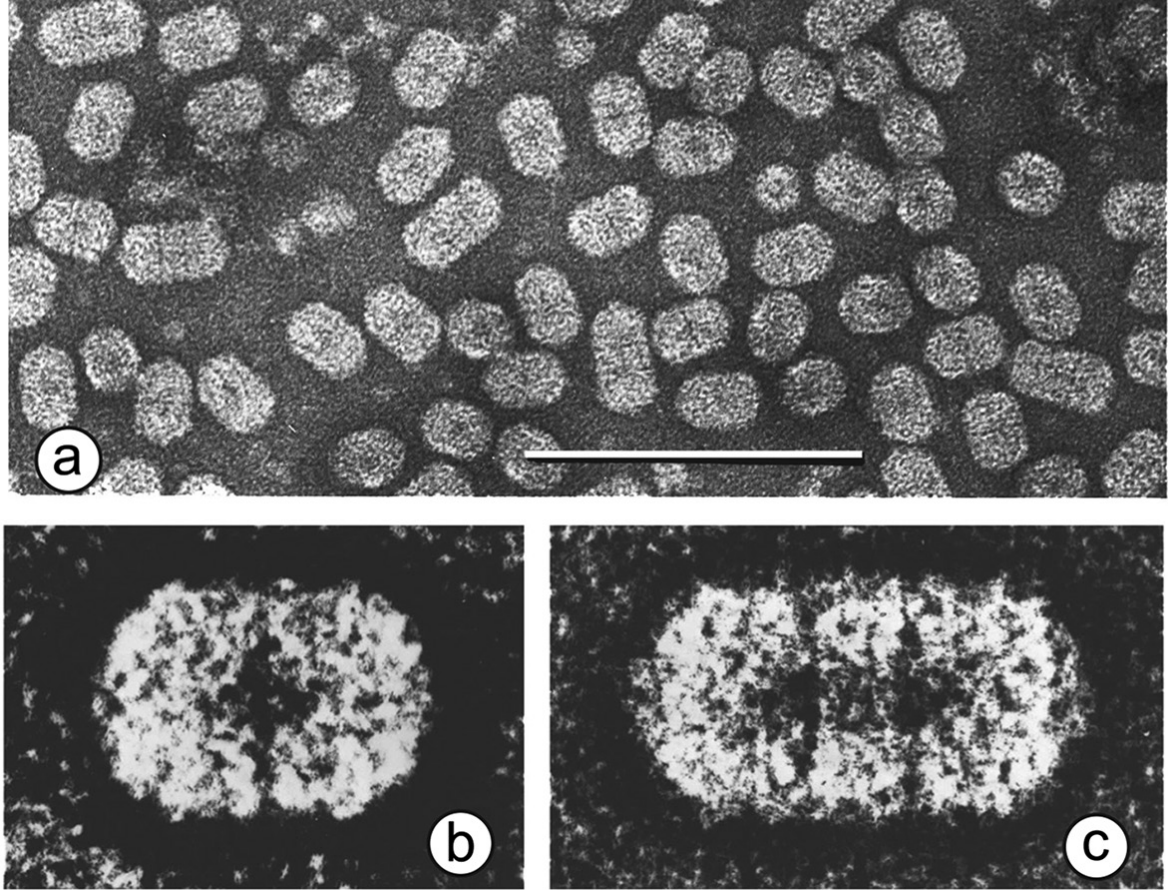
Virion morphology: (a) negative-contrast electron micrographs (uranyl acetate) of purified particles of Ourmia melon virus (bar, 100 nm); (b, c) features of the two commonest particle types (two- and three-disc), enhanced by photographic superimposition.

Members of the genus *Ourmiavirus* are plant viruses with non-enveloped bacilliform virions that are composed of a single 23.8 kDa coat protein. Electron microscopy reveals particles with conical ends (apparently hemi-icosahedral) and cylindrical bodies that are 18 nm in diameter (Table 1, Fig. 1). Most particles consist of two discs (giving a particle length of 30 nm), with other particles having three (37 nm) or, more rarely, either four (45.5 nm) or six discs (62 nm). Members of other genera infect fungi and are not encapsidated.

**Table 1.** Characteristics of members of the family *Botourmiaviridae*

Typical member:	Ourmia melon virus VE9 (RNA1: EU770623; RNA2: EU770624; RNA3: EU770625), species *Ourmia melon virus*, genus *Ourmiavirus*
Virion	Bacilliform (18×30–62 nm) with a 23.8 kDa coat protein (*Ourmiavirus*) or unencapsidated (members of other genera)
Genome	Positive-sense RNA of 2–3 kb (*Botoulivirus*, *Magoulivirus* and *Scleroulivirus*) or three segments of 2.8, 1.1 and 0.97 kb (*Ourmiavirus*)
Replication	Cytoplasmic; virion assembly is coupled to active replication
Translation	From genomic RNA; each genomic segment is monocistronic
Host range	Plants and fungi
Taxonomy	Realm *Riboviria*, kingdom *Orthornavira*, phylum *Lenarviricota*, class *Miaviricetes*, order *Ourlivirales*, family *Botourmiaviridae*, several genera each with multiple species

## Genome

**Fig. 2.**
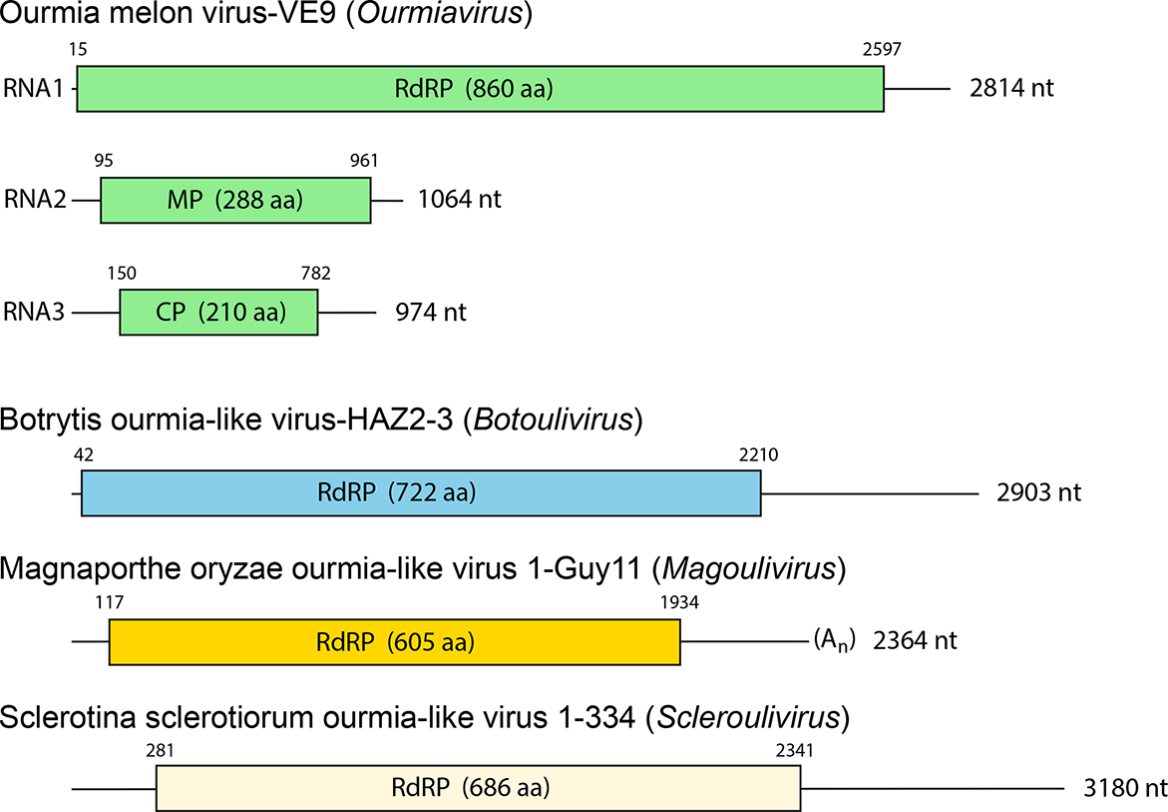
Genome organization of representative isolates of the family *Botourmiaviridae*. Boxes indicate the position and size of the ORFs encoding the coat protein (CP), movement protein (MP) and RNA-directed RNA polymerase (RdRP).

The genome of ourmiaviruses consists of three segments of positive-sense, ssRNA (2814, 1064 and 974 nt for Ourmia melon virus) [1] encoding an RNA-directed RNA polymerase (97.5 kDa; RNA1), a movement protein (31.6 kDa; RNA2) and a coat protein (23.8 kDa; RNA3) (Fig. 2) [2]. Members of other genera have a genome with a single segment of 2000–3200 nt encoding an RNA-directed RNA polymerase [3–6]. Unusually for the family, the genome of Magnaporthe oryzae ourmia-like virus 1 (species *Magnaporthe magoulivirus 1*, genus *Magoulivirus*) is polyadenylated at the 3′-end (4).

## Replication

Ourmiavirus replication is dependent on the virus RNA-directed RNA polymerase. Synthesis of the ourmiavirus coat protein from actively replicating RNA3 is necessary for both virion assembly and systemic infection of the host [2]. The ourmiavirus movement protein determines symptoms and forms tubular structures involved in cell-to-cell movement [7] and may undergo post-translational modification. Botoulivirus, magoulivirus and scleroulivirus replication is strictly dependent on the virus RNA-directed RNA polymerase.

## Pathogenicity

Members of the genera *Botoulivirus*, *Magoulivirus* and *Scleroulivirus* infect fungi from the genera *Botrytis*, *Magnaporthe* or *Sclerotinia*, respectively [3–5]. Members of the genus *Ourmiavirus* infect plants; Ourmia melon virus infects melon, producing chlorotic spots and irregular ringspots [8], Epirus cherry virus produces rasp-leaf symptoms in cherry, and cassava virus C induces severe stunting and a yellow mosaic pattern in cassava.

## Taxonomy

Botourmiaviruses are more closely related to members of the genus *Narnavirus* (family *Narnaviridae*) than to members of the genus *Mitovirus* (family *Narnaviridae*). Members of different botourmiavirus genera differ by >70 % in RNA-directed RNA polymerase amino acid sequence.

## Resources

Current ICTV Report on the family *Botourmiaviridae*: ictv.global/report/botourmiaviridae
